# Eye Movement Compensation and Spatial Updating in Visual Prosthetics: Mechanisms, Limitations and Future Directions

**DOI:** 10.3389/fnsys.2018.00073

**Published:** 2019-02-01

**Authors:** Nadia Paraskevoudi, John S. Pezaris

**Affiliations:** ^1^Brainlab – Cognitive Neuroscience Research Group, Department of Clinical Psychology and Psychobiology, University of Barcelona, Barcelona, Spain; ^2^Institute of Neurosciences, University of Barcelona, Barcelona, Spain; ^3^Department of Neurosurgery, Massachusetts General Hospital, Boston, MA, United States; ^4^Department of Neurosurgery, Harvard Medical School, Boston, MA, United States

**Keywords:** gaze contingency, visual prosthetics, artificial vision, eye movements, blindness, neuroprosthetics

## Abstract

Despite appearing automatic and effortless, perceiving the visual world is a highly complex process that depends on intact visual and oculomotor function. Understanding the mechanisms underlying spatial updating (i.e., gaze contingency) represents an important, yet unresolved issue in the fields of visual perception and cognitive neuroscience. Many questions regarding the processes involved in updating visual information as a function of the movements of the eyes are still open for research. Beyond its importance for basic research, gaze contingency represents a challenge for visual prosthetics as well. While most artificial vision studies acknowledge its importance in providing accurate visual percepts to the blind implanted patients, the majority of the current devices do not compensate for gaze position. To-date, artificial percepts to the blind population have been provided either by intraocular light-sensing circuitry or by using external cameras. While the former commonly accounts for gaze shifts, the latter requires the use of eye-tracking or similar technology in order to deliver percepts based on gaze position. Inspired by the need to overcome the hurdle of gaze contingency in artificial vision, we aim to provide a thorough overview of the research addressing the neural underpinnings of eye compensation, as well as its relevance in visual prosthetics. The present review outlines what is currently known about the mechanisms underlying spatial updating and reviews the attempts of current visual prosthetic devices to overcome the hurdle of gaze contingency. We discuss the limitations of the current devices and highlight the need to use eye-tracking methodology in order to introduce gaze-contingent information to visual prosthetics.

## Introduction

Over the last few years, there has been a growing interest in stimulating different parts of the visual processing stream including the retina, the optic nerve, the lateral geniculate nucleus (LGN) or the primary visual cortex (Pezaris and Eskandar, 2009). However, the complexity of the visual system and the interaction between visual and oculomotor processes have been found to pose significant difficulties when attempting to provide artificial percepts similar to those of natural vision (Brindley and Lewin, 1968; Burr, 2004; Pezaris and Eskandar, 2009; Hafed et al., 2016). By exploring how the brain makes sense of the visual information, visual neuroscience can provide valuable insights to artificial vision studies. Thus, in addition to its importance for basic research, the goal to gain a better understanding of visual perception is also relevant to recent efforts that aim to restore vision in blind individuals.

The challenge in designing visual prostheses is not limited to manufacturing such devices. One of the most crucial and important challenges is to introduce designs that efficiently interface with the visual brain. Despite the rapid evolution of microtechnology, many of these devices fail to update visual information as the patients move their eyes to interact with the environment. This shortcoming complicates the creation of percepts in world-centered coordinates, which highly depends on conveying information to the brain that is associated with the correct location in the visual scene. Inspired by the need to overcome this hurdle, this review aims to address gaze contingency as one of the major challenges for artificial vision by presenting both its neural substrate and the means current prosthesis projects include gaze-contingent information in their studies. Finding new ways to mimic the normal oculomotor and visual function is important in order to develop prostheses that could benefit blind individuals in their mobility and navigational performance, while also increasing their independence when performing activities of daily living (ADL).

## Spatial Updating

Motion is an inherent aspect of human life. We move our arms, legs and body in order to navigate in our environment. Most importantly, even in the absence of whole-body movements, we move our eyes to capture objects of interest and scan the world around us. These eye movements cause the visual representation of objects in our world to move across our retinas (Burr, 2004; Klier and Angelaki, 2008; Inaba and Kawano, 2016). With each new eye movement, a given location in the world is projected to a new location on the retina (Burr, 2004; Heiser and Colby, 2006; Klier and Angelaki, 2008). And yet, despite these frequent displacements, the visual brain is able to create a stable and continuous mental image of the external world by compensating each retinal snapshot with the gaze direction used to make it (Klier and Angelaki, 2008; Pezaris and Eskandar, 2009; Rao et al., 2016).

**FIGURE 1:**
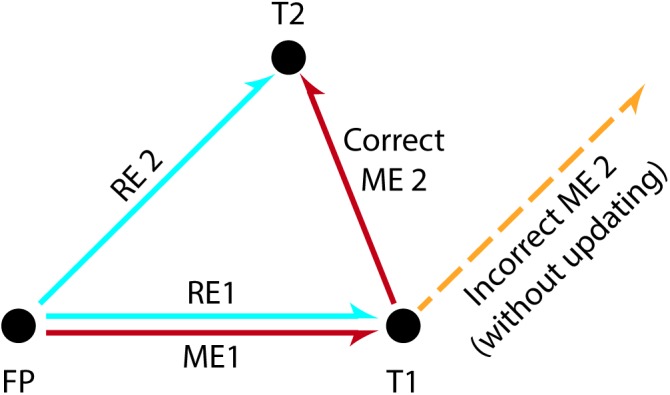
The double-step saccade task used to illustrate spatial updating. Subjects fixate a centrally presented stimulus (FP) and subsequently they are asked to fixate two successively and briefly presented stimuli at different screen locations (T1, T2). The perceptual distance between the fovea and each target at the time of stimulus presentation is called the ***retinal error*** (“error” because the value must be brought to zero in order to achieve the goal of foveation). The eye movements required to correctly foveate each target are in turn called the ***motor errors.*** To make a saccade to the first target T1, the motor error ME1 can be deduced directly from the retinal error RE1 for T1 and therefore correctly executed. However, the first saccade to T1 displaces T2 from the location where T2 initially appeared on the retina. Thus, executing a second saccade based purely on the originally observed retinal error RE2 would lead to a failed attempt to foveate T2 (orange dashed line). Instead, the motor plan ME2 for T2 needs to compensate for the intervening saccade to T1. This is accomplished by subtracting RE1 from RE2. Recall that both T1 and T2 are only briefly presented, and are extinguished prior to the execution of eye movements. (Adapted from Mays and Sparks, 1980 and Klier and Angelaki, 2008).

There must be a mechanism supporting the perceptual stability of a visual scene, raising the question of how the percepts resulting from each retinal displacement are updated in the appropriate coordinates and integrated into a whole. For example, to correctly fixate two subsequently presented visual stimuli at different screen locations, the information about the intervening eye movement caused by the gaze shift toward the first stimulus needs to be taken into account in order to correctly localize the second stimulus (see Figure 1). This observation suggests that the brain must combine different kinds of information: the retinal signal caused by the position of the stimulus on the retinal surface (i.e., *retinal error*; see section “Glossary” for this and other terms) and the information about the amplitude and direction of the intervening saccade toward the previous stimulus (i.e., *motor error*; Klier and Angelaki, 2008). Widely known as *spatial updating* (Duhamel et al., 1992; Klier and Angelaki, 2008) or the *saccadic displacement problem* (Inaba and Kawano, 2016), this process combines retinal signals with *extra-retinal information* so as to create spatial constancy, which in turn provides us with a continuous, stable representation of visual space (Klier and Angelaki, 2008). This ability enables us to reach objects accurately and interact with them effectively, which makes spatial updating highly relevant for visual prosthesis development.

Over the last decades, there has been a growing interest in investigating the mechanisms that mediate spatial updating. To this end, most studies have used the double-step saccade task, as first introduced by Hallett and Lightstone (1976a, b; see Figure 1). During the generalized version of this task, subjects are instructed to fixate a target (FP) at the center of the screen while two peripheral visual stimuli are successively flashed (e.g., T1 followed by T2). Subjects are then asked to make a saccade toward the first stimulus (FP→T1) and subsequently make a second saccade toward the second stimulus (T1→T2). However, for the second saccade to be accurate (i.e., to correctly localize T2), the brain must take into account both the amplitude and direction of the eye movement toward the first stimulus (i.e., the motion vector for the first target). Extensive use of this task in electrophysiological studies has contributed to determining the crucial mechanisms underlying spatial updating (Westheimer, 1954; Hallett and Lightstone, 1976a,b; Becker and Jüergens, 1979; Mays and Sparks, 1980; Zimmermann et al., 2015, 2018).

**FIGURE 2:**
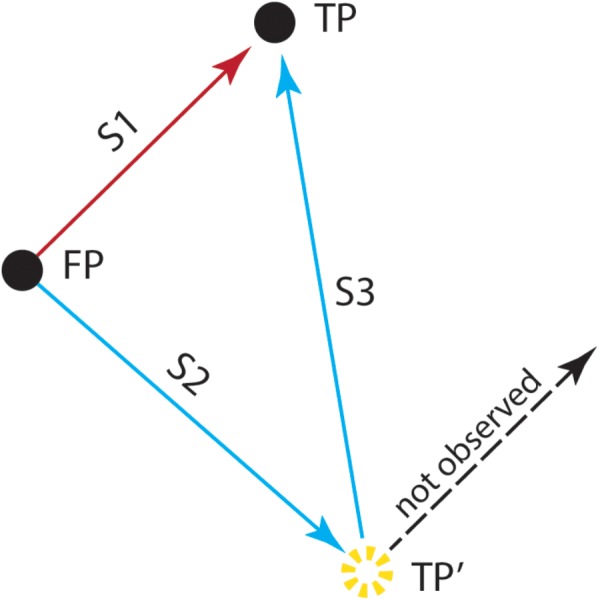
The double-step saccade task used by Sparks and Mays (1983). After fixating a stimulus presented at the center of the screen (FP), the monkey was trained to generate an eye movement (S1) to the location of the target (FP→TP). However, in some trials and after the target’s offset, the animal received a train of electrical stimulation to the superior colliculus, before initiating the eye movement. This stimulation drove the animal’s eyes away from the fixation target (S2) to another position (FP→TP′). To generate an accurate saccade (S3) to the remembered target location (TP′→TP), the animal had to take into account the amplitude and direction of the stimulation-induced intervening eye movement. This study showed that the electrically induced saccade (S2) was followed by a saccade (S3) toward the location of the target stimulus (TP), which allowed the animal to correctly localize the target (TP). Saccades that did not take into account the electrically induced perturbation (dashed line) were not observed, demonstrating that the perception of TP occurred in spatial coordinates that are deduced from a combination of retinal activity and eye position. (Adapted from Sparks and Mays, 1983).

At the neural level, spatial updating seems to be mediated by RF locations that shift as our gaze moves to different locations (Klier and Angelaki, 2008). The first evidence showing that retinal signals are combined with information about the gaze position at a given instance (an example of *extra-retinal* information) came from the seminal double-step saccade study of Sparks and Mays (1983; see Figure 2). In a typical trial, after fixating a central target, the monkey had to generate an eye movement to the location of a peripheral target that was briefly flashed. However, in 30% of the trials, after the target disappeared, but before the monkey could initiate the saccade, a train of electrical stimulation was delivered to the superior colliculus (SC). This stimulation drove the animal’s eyes away from the fixation target to another position in the orbit, so that then, in order to generate an accurate saccade to the remembered target location, the animal had to take into account the amplitude and direction of the stimulation-induced intervening eye movement. This methodology allowed them to test whether the electrically evoked saccade would affect the characteristics of the subsequent naturally made saccade toward the target. The results were straightforward: The monkey’s final eye position was at the approximate location of the target, that is, the electrically induced saccade was followed by a saccade that had been corrected for the perturbation. This finding indicates that neurons in SC are responsible for recomputing the motor error resulting from intervening eye movements, thereby providing strong evidence supporting the idea that retinal signals are combined with information about instantaneous eye position.

Similar findings have been reported in other areas as well such as frontal eye field (FEF) and the lateral intraparietal area (LIP) (for a review see Rao et al., 2016). It has been shown that neurons in FEF responded to stimuli presented as targets for second saccades in double-saccade tasks, although these stimuli were not shown in their unadapted RF (Bruce and Goldberg, 1985; Bruce et al., 1985). This effect is also present in LIP (Heiser and Colby, 2006), which shares reciprocal functional connectivity with FEF (Blatt et al., 1990; Stanton et al., 1995; Chafee and Goldman-Rakic, 1998, 2000; for a review see Rao et al., 2016). Single neurons in LIP have been found to respond in a predictive manner to anticipate what the visual scene would look like after a saccade (Heiser and Colby, 2006). These neurons seem to play a crucial role in spatial updating, with most studies reporting LIP activity when a saccade shifted the RF onto a previously stimulated location (Andersen and Mountcastle, 1983; Andersen et al., 1990, 1993; Goldberg and Bruce, 1990; Duhamel et al., 1992).

**FIGURE 3:**
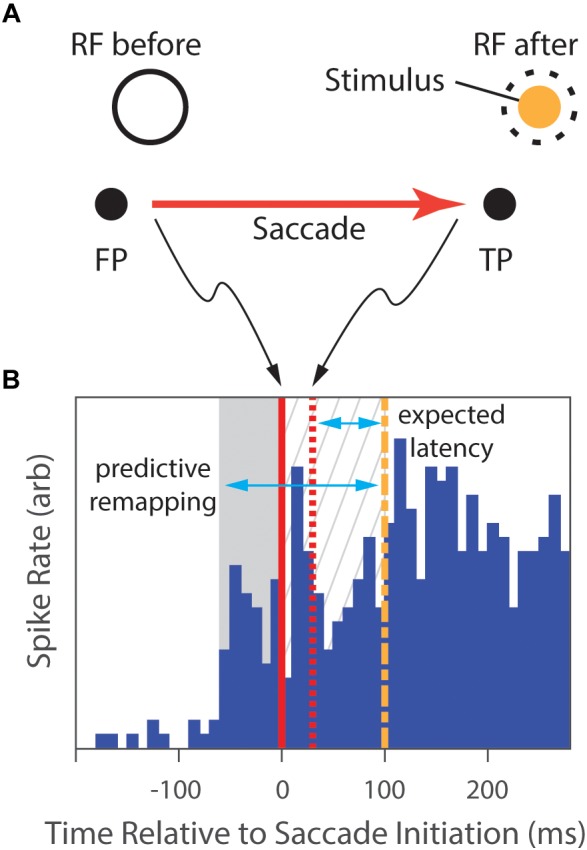
The predictive-remapping task from Duhamel et al.’s study (1992). **(A)** The subject fixated a central point (FP), placing the RF of the cell under study on a blank part of the screen (RF before). Then, a target point (TP) to which the animal was required to make a saccade was presented simulatenously with a peripheral visual stimulus (Stimulus) in the future, post-saccade, RF location of the neuron (dashed circle). **(B)** During initial fixation, there is no neural response (blue histogram). However, slightly preceding the initiation of the saccade (solid red line), the cell begins to fire (gray background). Saccades typically completed in 30 ms (dashed red line), placing the classical RF over the Stimulus. With normal latency, the response would be expected to start 75 ms later (dashed green line), but the cell has continued to respond in the meanwhile (gray hatched background). We expect that, as the animal shifs its gaze to the locus of the target, the RF shifts as well, and the cell would begin to fire after the normal response latency following the saccade completion. However, portions of the discharge of the cell not only preceded that expected latency (gray hatched background), but also preceded the saccade (gray area), suggesting that the location of the RF shifted to accurately anticipate the position after the eye movement. (Data extracted from Duhamel et al., 1992, especially figure 2b).

Inspired by these findings, Duhamel et al. (1992) were the first to demonstrate the ability of these LIP neurons to use information about intended saccades to update the neural representation of the visual scene (see Figure 3). After mapping each neuron’s RF, they had the animal fixate a central point, and then presented a peripheral target to which the animals had to initiate a saccade. During the animal’s fixation at the first location, a visual stimulus was presented in the future, post-saccade, RF location of the neuron. As the animal shifted its gaze to the locus of the second target, the RF shifted as well, and the cell began to fire. Surprisingly, the discharge of the cell *preceded* the saccade, indicating that the location of the RF shifted before the onset of the eye movement. Notably, 44% of the LIP neurons recorded in this study were found to anticipate the retinal consequences of the intended saccades, thus indicating that cells in parietal cortex had *a priori* knowledge of the imminent onset, magnitude and direction of saccades (Duhamel et al., 1992; Rao et al., 2016). But, what is the source of this information?

This information has been suggested to come from a motor efference copy or corollary discharge, i.e., copies of “voluntary, outgoing motor commands” (Klier and Angelaki, 2008; Rao et al., 2016) representing eye position. For example, in order to change our gaze, a neural command must be generated and then sent to the motor neurons of the brainstem that are responsible for controlling the eye muscles. A copy of this motor command could then be sent to visual mapping areas and can be, subsequently, used by the brain for several tasks and processes, one of them being spatial updating (Klier and Angelaki, 2008; Caspi et al., 2017). While direct evidence for an efference copy has not yet been identified, the mounting indirect evidence is substantial, with most studies pointing to sub-cortical and extra-striate areas (Heiser and Colby, 2006; Sommer and Wurtz, 2006; Inaba and Kawano, 2013; Rao et al., 2016). Specifically, SC and FEF have been implicated as candidate areas responsible for generating and supplying a copy of the eye movement command to LIP (Heiser and Colby, 2006), while additional evidence suggests that the corollary discharge mechanism may result from the operations of the pathway from SC to mediodorsal thalamus (MD) to FEF (Sommer and Wurtz, 2006).

Converging evidence has attributed the RF shifts to a *predictive remapping* mechanism that contributes in maintaining visual stability. According to the predictive remapping account, cells in several brain areas exhibit a transient change of their RF location immediately before the initiation of a saccadic eye movement (Duhamel et al., 1992; Inaba and Kawano, 2016). Supporting data comes from studies showing that presaccadic remapping occurs in both cortical and subcortical areas (e.g., V1, V2, V3, V3A: Nakamura and Colby, 2002; V4: Neupane et al., 2016a,b; SC: Walker et al., 1995; Churan et al., 2012; Daddaoua et al., 2014; FEF: Umeno and Goldberg, 1997, 2001; Sommer and Wurtz, 2006; Mayo et al., 2016; LIP: Heiser and Colby, 2006, but not in MT: Inaba and Kawano, 2016; for reviews see Wurtz et al., 2011; Rao et al., 2016), whose neurons transiently shift their RFs to the location into which the saccade brings the stimulus (Duhamel et al., 1992; Inaba and Kawano, 2016; Rao et al., 2016) and then shift it back to the original (retinotopic) location as shown by its continued firing to the stimulus (Duhamel et al., 1992; Wurtz et al., 2011; Inaba and Kawano, 2016; Rao et al., 2016). This presaccadic remapped response differs from the normal visual response in two important aspects. First, its spatial location depends on the initial RF, as well as on the vector of the subsequent saccade. Second, the visual latency of a given neuron does not affect the timing of the remapped response (Rao et al., 2016). Rather, the neuron’s response correlates with the saccade onset, indicating that instead of being time-locked to the neuron’s visual latency, the remapped responses during RF shifts are synchronized with the saccadic motor act (Sommer and Wurtz, 2006, 2008). The temporal alignment between RF shifts and saccade onset further supports the hypothesis implicating a corollary discharge signal as the underlying cause for the RF shifting.

The second candidate mechanism to explain this remapping phenomenon is known as spatiotopic representation or the *gain field* account (Andersen and Mountcastle, 1983; Andersen et al., 1990, 1993; Cassanello and Ferrera, 2007; Inaba and Kawano, 2016). The gain field framework holds that spatiotopic representation is mediated through neurons whose visual responses are multiplicatively modulated by eye position (Andersen and Mountcastle, 1983; Andersen et al., 1990, 1993; Cassanello and Ferrera, 2007). In other words, the firing frequency of gain field neurons increases or decreases as if it were being multiplied by gaze angle, scaled by some gain factor, while the shape and the location of their RF remains unaffected by gaze position (Andersen and Mountcastle, 1983; for a review see Blohm and Crawford, 2009). Contrary to the eye-centered representations of retinal neurons, the gain-modulated responses observed in parietal regions indicate that visual images in higher-order brain areas are represented in spatiotopic, rather than retinotopic, coordinates (Andersen and Mountcastle, 1983; Andersen et al., 1990; Andersen et al., 1993; Salinas and Abbott, 2001; Cassanello and Ferrera, 2007). This finding has led researchers to propose this mechanism as being responsible for combining retinal information with eye position signals (Klier and Angelaki, 2008). This combining, in turn, allows the forming of head-centered target representations, which are necessary for object localization, motor execution, and visuomotor coordination (Salinas and Abbott, 2001).

Neurons whose visual responses are modulated by gaze position (i.e., gain field neurons) were first found in area 7a and then in several other extrastriate visual areas, such as LIP, MST, MT, VIP (Andersen and Mountcastle, 1983; Andersen et al., 1990; Andersen et al., 1993; Crespi et al., 2011; Inaba and Kawano, 2016). In an important study, Andersen et al. (1990) examined the effect of eye position on light-sensitive, memory, and saccade-related neural activity in two cortical areas (i.e., LIP and 7a). They employed a memory saccade task, as first introduced by Hikosaka and Wurtz (1983) and later developed by Andersen and colleagues (e.g., Gnadt and Andersen, 1988; Andersen and Gnadt, 1989; Andersen et al., 1990), in order to dissociate the visual, memory, and motor-related responses. During the initial *baseline period*, the animals fixated at one of 9 fixation points. Subsequently, an eccentric saccade target was presented for 300 ms (the *light-sensitive period*). Once the target disappeared, the animal had to withhold its saccadic response and remember the target’s location (the *memory period*). Finally, the animal had to initiate a saccade toward the remembered location (the *saccade period*). Neural direction tuning was determined by measuring responses during each period for targets around a circle. The effect of eye position on all three responses for this direction was then tested so as to examine gain fields, i.e., the variation in neural response as a function of eye position when all other parameters were held constant. They found that both LIP and 7a neurons yielded significant responses for all three types of activity (light-sensitive, memory, and saccade), with the majority of the cells exhibiting a tonic background activity closely linked to eye position. Although direction tuning remained unaffected by eye position, the magnitude of the response was influenced, revealing a modulatory role of eye position in determining these three response types in both cortical areas. Taken together, these findings indicate that LIP and 7a neurons display gaze-dependent activity, which, operating simultaneously at different processing stages, could possibly generate a large final effect (Salinas and Abbott, 2001).

Although motor efference copies and gain fields have been proposed as the mechanisms underlying visual mapping in sighted individuals, it remains unknown whether these processes continue to operate in the same fashion with artificial vision in blind individuals (Caspi et al., 2017). As current prosthetic devices do not provide foveal vision, the question of whether implanted individuals maintain the ability to employ these mechanisms to achieve visual stability has not been addressed (Caspi et al., 2017). Evidence shows that only attended stimuli exhibit spatiotopic tuning (Crespi et al., 2011), suggesting that this mechanism may not operate normally on blind patients that lack a functioning fovea. However, patients suffering from natural central vision loss have been found to use extrafoveal areas as foci of attention (*saccadic rereferencing*) in order to efficiently commit an object into long-term memory (Geringswald et al., 2015). Surprisingly, this flexible deployment of attention was not evident in sighted individuals that took part in a study that simulated a central scotoma (Geringswald et al., 2016). Contrary to patients with central vision loss, sighted individuals were not able carry out all visual processing with peripheral vision, and exhibited impaired visual long-term memory for everyday objects in natural scenes, suggesting that saccadic rereferencing may require several hours of training before the oculomotor system develops a stable extrafoveal preferred retinal locus for fixation (Chung, 2013; Kwon et al., 2013; Walsh and Liu, 2014). Overall, this remarkable flexibility of the oculomotor system and the efficient deployment of peripheral vision contribute to maintaining normal visual memory function (Kwon et al., 2013; Geringswald et al., 2015, 2016), which could potentially allow the use of perceptual learning paradigms in order to develop alternative rehabilitative strategies for people with central vision loss. Taken together, these findings highlight that the effects of attention and saccadic rereferencing are highly relevant for understanding the strategies adopted by the visual brain to compensate for sensory loss, which may have several implications for artificial vision projects.

Until recently, the predictive remapping and gain field mechanisms have been considered as different – and contra dicting – explanations to the saccadic displacement problem (Andersen et al., 1990, 1993; Duhamel et al., 1992; Inaba and Kawano, 2016). The two mechanisms differ in three important aspects: (a) the method of retinotopic-to-spatiotopic transformation (translation and changes in RF locations vs. multiplicative gain of RF response as postulated by the predictive remapping and gain field frameworks, respectively), (b) the locus of the effect (both cortical and subcortical areas vs. higher-order parietal areas), and (c) the duration of the effect (transient change of RF profile vs. persistent spatiotopic mapping). However, given that some areas (e.g., LIP) have been implicated in both frameworks, it is important to test for possible interactions between the two mechanisms in single neurons. Indeed, gaze angle has been recently found to modulate the visual sensitivity of neurons in medial superior temporal area (MST) after saccades both to the currently presented visual stimuli and to the visual memory traces remapped by the saccadic movements (Inaba and Kawano, 2016). These dual responses suggest that the two mechanisms act cooperatively in MST neurons and may both play a crucial role in providing a coherent representation of a continuous and stable visual scene.

## Gaze Compensation in Artificial Vision

Since the late 1960s, there has been accelerating interest in the development of visual prostheses (Schiller and Tehovnik, 2008; Pezaris and Eskandar, 2009; Chuang et al., 2014; Goetz and Palanker, 2016), fueled in part by the success of cochlear implants and in part by improvements in microelectronics (Shepherd et al., 2013). Although this field is far from mature, recent progress on several fronts has been rapid, embolding predictions of restoring high-quality vision in the near future (e.g., Lorach et al., 2015). While many groups worldwide are currently working on the development of visual prosthesis, only a few prostheses have currently received FDA approval or CE marking (Zrenner, 2002; Zrenner et al., 2010, 2017; Stingl et al., 2013a,b, 2015; Chuang et al., 2014; Edwards et al., 2018): Alpha IMS and Alpha AMS (first-, and second-generation devices, respectively, Retina Implant AG, Reutlingen, Germany), Argus II (Second Sight Medical Products Inc., Sylmar, CA, United States), PRIMA and IRIS II (Pixium Vision, Paris, France). However, as discussed in the following sections, the visual percepts provided by these devices are, to date, not sufficient to yield substantial improvements in patients’ life, and thereby to enhance their performance in ADL.

Although the commercially available devices described above are placed in the early stages of the visual pathway (i.e., retina; see Table 1), other locations of the visual processing chain have been also proposed for potential stimulation for visual prostheses (e.g., LGN; Pezaris and Reid, 2007, 2009; Pezaris and Eskandar, 2009; visual cortex; Brindley and Lewin, 1968; Dobelle, 2000; Schiller and Tehovnik, 2008; Lewis et al., 2015). Discussions on the advantages and disadvantages of each approach highlight that multiple factors need to be considered before proposing one area for stimulation over another: potential spatial arrangements of underlying representations and realizable phosphene layout, the stability of the implant in a given area, the effects of stimulation on neural tissue, the surgical risks, and the risks of long-term infection (Pezaris and Eskandar, 2009; Goetz and Palanker, 2016). Given that eye movements modulate visual processing in the brain (Leopold and Logothetis, 1998; Hafed and Krauzlis, 2010; Hafed et al., 2016), optimize eye position during fixation (Hafed et al., 2009; Kagan and Hafed, 2013), and prevent retinal fading (Coppola and Purves, 1996), we propose an additional factor for consideration: whether or not current prosthetic designs feature gaze compensation. In the following sections we will review the mechanisms for integrating gaze contingency into a visual prosthesis design, by categorizing the current devices based on their design and the technology they are using (Shepherd et al., 2013). Most current devices include an external video camera, a video processing unit, a power supply, a transcutaneous telemetry link, an implantable stimulator and an electrode array located either at the retina or later stages of the visual pathway (Ahuja et al., 2011; Zhou et al., 2013; Chuang et al., 2014; Caspi et al., 2017). In visual prosthetics with head-mounted cameras, patients have to learn to hold their eyes fixed and use relatively coarse head movements for visual search. Alternatively, instead of an external camera, some research groups have used an array of photosensitive circuits (typically photodiodes) implanted within the eye that utilize the existing ocular optics and oculomotor plant to localize a percept within the visual field (Zrenner, 2002; Shepherd et al., 2013; Stingl et al., 2013a,b, 2015; Lorach et al., 2015; Hafed et al., 2016). With these devices, the visual scene is, therefore, automatically updated on the intraocular imaging sensor by normal movements of the eye, which is why the latter approach has been suggested as a potential solution in providing world-centered visual representation (Zrenner, 2002; Shepherd et al., 2013; Stingl et al., 2013a,b, 2015; Lorach et al., 2015; Hafed et al., 2016). Thus, intraocular photovoltaic devices, (Zrenner, 2002; Stingl et al., 2013a,b, 2015; Boinagrov et al., 2014; Lorach et al., 2015), are not burdened with issues of gaze contingency.

**Table 1:** Summary of current retinal prosthetic devices.

Device	Company	Retinal location	Electrode number	Gaze contingency	Reference
STS	Nidek Co., Osaka/Gamagori, Japan	Suprachoroidal	49	no	Fujikado et al., 2016

BVA	Bionic Vision, Australia	Suprachoroidal	33	no	Ayton et al., 2014

Argus II	Second Sight Medical Products, CA, United States	Epiretinal	60	no	Ahuja et al., 2011; Caspi et al., 2017

Argus I	Second Sight Medical Products, CA, United States	Epiretinal	16	no	Yue et al., 2015

EPIRET3	EPIRET GmBH, Germany	Epiretinal	25	no	Menzel-Severing et al., 2012

IRIS II	Pixium Vision, France	Epiretinal	150	no	N/A

Alpha IMS	Retinal Implant AG, Germany	Subretinal	1500	yes	Stingl et al., 2015

Alpha AMS	Retinal Implant AG, Germany	Subretinal	1600	yes	Stingl et al., 2017

PRIMA	Pixium Vision, France	Subretinal	378	no	N/A

Boston Retinal Implant Project	Bionic Eye Technologies Inc., MA, United States	Subretinal	256	no	Kelly et al., 2013


### Retinal Approach

Most artificial vision research has been focusing on retinal implants that use external head-mounted cameras placed on the nose-bridge of glasses worn by the patient (see Table 1). These devices typically include an external video camera, a video processing unit, a power supply, a transcutaneous telemetry link, an implantable stimulator and an electrode array located at the suprachoroidal (STS, Bionic Vision Australia), subretinal (Boston Retinal Implant, PRIMA) or epiretinal (Argus I and II, EPIRET3, IRIS II) part of the retina (but a similar design is used in devices targeting later stages of the visual pathway; Ahuja et al., 2011; Chuang et al., 2014; Caspi et al., 2017; for reviews see Lewis et al., 2015; Yue et al., 2016). Based on the scene captured from the camera, the electrode array delivers a stimulation pattern so as to generate an artificial visual percept (Pezaris and Eskandar, 2009; Chuang et al., 2014), without adaptation for eye position, which is why with visual prosthetics with head-mounted cameras, patients must be trained to hold their eyes fixed and use scanning head motions to steer their gaze.

In the following sections, we will discuss the results obtained by studies assessing the efficacy of two devices that have been already tested in clinical trials (i.e., Argus II and Alpha IMS), as well as other efforts to restore vision via retinal stimulation (i.e., the latest version of Alpha IMS), as well as the Stanford approach (Boinagrov et al., 2014; Lorach et al., 2015).

#### Devices Using External Cameras

The Argus II retinal prosthesis stimulates the surviving retinal ganglion cells of RP patients with visual input from an external camera. Although external cameras do not require corneal or lens clarity (Chuang et al., 2014), the image stream acquired by the camera is determined by head position alone and not updated by eye position. Not surprisingly, changes in eye position have been found to affect the stimulation’s perceptual location of the Argus II (Caspi et al., 2017). This effect has led to instructing patients to maintain their eye position fixed in the forward position so as to align the pupillary and camera axes (Caspi et al., 2017), and to avoid large eye movements because of the perceived image displacements they cause. Holding the eyes fixed means that visual scanning is possible through head and body movements only, a somewhat unnatural and inefficient behavior when trying to accomplish ADL (Sommerhalder and Perez Fornos, 2017). It has been reported that Argus II-wearers exhibit improved orientation and mobility performance with the device on vs. off (Humayun et al., 2012), despite the awkwardness of its use, but, a close inspection of their results reveals that the performance improvements may be mainly due to the low task difficulty.

**FIGURE 4:**
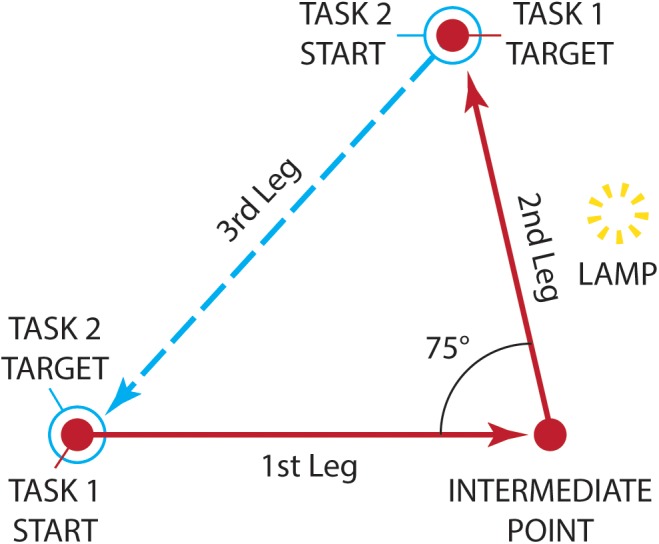
The navigational tasks employed by Garcia et al. (2015) as seen from above. Patients implanted with Argus II were initially guided along a path by the experimenter (solid line). The path comprised of an initial 2.5-meter leg, a rotation left, and a final 2-meter second leg. A lamp, acting as a visual landmark was placed midway along the second leg. For the path reproduction task (Task 1), participants were led to the start position (filled circle) and were asked to reproduce the path as accurately as possible (dashed line). For the triangle completion task (Task 2), participants started from the end of the reproduction path (open circle) and had to return directly to the initial start position (filled circle), thereby completing a walked triangle path (dotted line). (Diagram adapted from Garcia et al., 2015, Figure 2).

Indeed, the mobility-related improvements reported by Humayun et al. (2012) have been recently challenged by a study that examined whether Argus II-wearers benefit from the new visual signal together with non-visual self-motion information when navigating (Garcia et al., 2015). Based on evidence suggesting that sighted individuals can improve their navigational performance by integrating visual and vestibular or proprioceptive cues, it was initially hypothesized that if this device is indeed beneficial for the patients, they should exhibit the multisensory gain observed in controls when integrating different sensory signals. Participants took part in two navigational tasks, after having been guided along a path by the experimenter (see Figure 4). For the first task, *path reproduction*, participants were led to the start position and were asked to reproduce the path as accurately as possible. For the second task, *triangle completion*, participants had to then return directly to the start position, thereby completing a walked triangular path. However, the visual information provided by the device did not improve navigational performance as compared to trials where patients had to complete the same tasks with the device off. Specifically, while no improvements were observed during the path reproduction task when using the device, a multisensory benefit was found only for two out of four patients in the triangle completion task. Note that all four patients have reported that instead of using the device for navigation in everyday life, they adopted non-visual navigational strategies. The impediment for this particular device to assist the patients in performing daily activities has been primarily attributed to the low number of phosphenes and to the reduced visual field (Garcia et al., 2015; Stingl et al., 2015), but we speculate it could also be due to a lack of gaze compensation in the delivered image. Interestingly, the subjective reports by these four individuals about the frequency they used the device in their daily life (Garcia et al., 2015) are in agreement with the recent reports by Sommerhalder and Perez Fornos (2017) about the patients of the Argus II clinical trial: only one third used the device up to 18.9 h/week, another third up to 4.8 h/week, and crucially the last third less than 90 min/week.

#### Intraocular Light-Sensing Devices

The 1500-electrode Alpha IMS device (Retina Implant AG, Reutlingen, Germany) is a photovoltaic visual prosthesis that has been tested in multicenter clinical trials including 39 patients (Zrenner, 2002; Stingl et al., 2013a,b, 2015; Stronks and Dagnelie, 2014; Cheng et al., 2017; Zrenner et al., 2017). Similar to other photovoltaic approaches, the Alpha IMS subretinal implant directly converts light into electrical stimulation with relatively little processing. Importantly, the conversion takes place within the eye, which enables a naturally updated image to be delivered with each eye movement (Shepherd et al., 2013; Caspi et al., 2017). This approach more accurately mimics natural vision, supporting normal saccadic and smooth pursuit eye motions as well as prevention of image fading through microsaccades (Chuang et al., 2014; Hafed et al., 2016; Zrenner et al., 2017).

Despite being highly promising, especially with regard to gaze contingency, current versions of the Alpha IMS have some drawbacks. First, in contrast with Argus II where stimulation variables can be adjusted separately for each individual electrode, parameters in the Alpha IMS system (i.e., offset and gain, or equivalently brightness and contrast) have only single adjustments that affect all electrodes globally (Stingl et al., 2013a; Stronks and Dagnelie, 2014). Second, the Alpha IMS photodiodes and circuitry require an external power source and lead wire (Shepherd et al., 2013). The power transducer is implanted subdermally and charges wirelessly through a handheld control unit for adjusting brightness and contrast. A recent trial revealed corrosion of the hermetic seal and one severe adverse event (SAE) of subretinal bleeding with a subsequent increase in intraocular pressure (Stingl et al., 2013a), highlighting the need for further optimization of biocompatibility (Chuang et al., 2014) to improve the reliability and durability of the implant. It has been proposed to put the complete implant in a hermetically sealed housing out of metal or ceramics to protect the included electronics (Daschner et al., 2017). However, as light has to fall on the photodiodes, which are integrated with the stimulation electrodes that must be in direct contact with the retinal tissue, a traditional metal or ceramic hermetic housing is not directly viable.

The issue of longevity has been addressed with the new generation implant, the 1600-electrode Alpha AMS, which has received CE mark in March 2016 and is being tested in a new trial in a cohort of 15 patients (Daschner et al., 2017; Maclaren, 2017; Stingl et al., 2017; Zrenner et al., 2017; Edwards et al., 2018). Promising results have been reported for the expected median lifetime of the new implant (i.e., 3.3 years for Alpha AMS as compared to the 0.6 median lifetime reported for Alpha IMS). Although interim results report that the implant-mediated visual perception was stable in most of the implanted patients (e.g., 7 out of 15 patients and 5 out of 6 patients) over an observation period of 12 (Stingl et al., 2017) and 24 months (Edwards et al., 2018), respectively, it remains to be seen whether the longevity of the new version has been indeed considerably improved.

The most important limitation, though, is that although Alpha IMS consists of a 1500 photodiode array, visual acuity remains unexpectedly poor and clinical outcomes are highly inconclusive (for a review see Zrenner et al., 2017). Simulation studies from other groups have demonstrated that 500 distinct phosphenes can provide useful visual information in letter recognition and reading tasks (Sommerhalder et al., 2004; Kyada et al., 2017), as well as in navigational, mobility and visuomotor coordination tasks (Perez Fornos et al., 2008). Thus, given the high phosphene density offered by Alpha IMS, one would expect improved functional outcomes in patients using the device. Stingl et al. (2013a) assessed visual acuity in 9 patients using the standardized Landolt C-rings test. They reported visual acuity in 2 of 9 subjects only, with measurements of 20/2000 and 20/546 of Snellen acuity that correspond to logMAR 2.00 and 1.43, respectively (Stingl et al., 2013a). Previous studies with patients implanted with Alpha IMS reported visual acuity of maximum 1.69 logMAR, which corresponds to 0.816 phosphenes per degree (Zrenner et al., 2010). However, the implant itself had 2.98 electrodes per degree (Eiber et al., 2013), thus it would have been expected to provide a better visual acuity.

One of the most recent studies of this group assessed the influence of implant eccentricity (i.e., position of the implant in relation to the fovea) on functional outcomes (Stingl et al., 2013b). In eight patients, the implant was placed subfoveally (directly behind the fovea), while in the other ten the placement was parafoveal (near the fovea, but not directly behind it). Among patients with parafoveal placement of the implant, 80% (8/10) could perceive light, 10% (1/10) recognized location, and 10% (1/10) correctly distinguished stripe patterns up to a resolution of 0.33 cycles/degree. Additionally, all patients in this group failed to pass motion-detection or Landolt C-ring tests. However, when the implant was placed subfoveally, 100% of patients passed light perception and localization tests (8/8), 75% (6/8) of them could resolve motion up to 35 degrees/s, and 88% (7/8) were able to correctly distinguish stripe patterns up to a resolution of 3.3 cycles/degree. Yet, despite high-density photocells and a subfoveal placement of the implant, only 38% (3/8) passed a Landolt C-ring test, demonstrating Snellen visual acuities of 20/2000 (logMAR 2.0), 20/950 (logMAR 1.68), and 20/546 (logMAR 1.44), as in Stingl et al. (2013a). Note, though, that 20/500 is needed for normal reading without visual aids (Trauzettel-Klosinski, 2009). More importantly, despite being the best-reported acuity to date, it is still well below the legal blindness limit (logMAR 1.0; Snellen 20/200). The theoretical maximum acuity, 1500 pixels would be 20/333-20/250 (Zrenner et al., 2017), which is far better than the best measurement reported (Stingl et al., 2013a,b). These disappointing results have been suggested to be caused by cross-talk between adjacent electrodes or from the extent to which the activation of a single electrode depends on the activation of the neighboring electrodes on the array (Wang et al., 2012; Eiber et al., 2013; Stronks and Dagnelie, 2014; Yue et al., 2016).

Similar to the Alpha IMS and AMS devices, the Stanford approach also uses a photosensitive array to restore visual function (Boinagrov et al., 2014; Lorach et al., 2015). Although the illumination levels required to achieve stimulation are above normal visual exposure, the photocells are efficient enough that IR exposure is still within safety limits.

Stimulation through this photovoltaic prosthesis has been reported to yield similar retinal and cortical responses to those from natural illumination of the healthy retina (Lorach et al., 2015). Responses to both forms of stimulation have been found to exhibit adaptation for static images, high-frequency flicker fusion, and nonlinear spatial summation that are well-studied features of normal vision. Recordings of visually evoked potentials indicated that visual acuity was significantly better than the acuity reported in clinical trials with patients implanted with either a camera-mounted prosthesis (Humayun et al., 2012) or intraocular light-sensing devices (Stingl et al., 2013a,b). This improvement might result from the low levels of cross-talk between adjacent electrodes due to the tight confinement of the electric field (Lorach et al., 2015), which would be expected to increase contrast and spatial resolution of the stimulation. Overall, the Stanford approach has several advantages compared to the other retinal approaches, with the most important one for this review being the incorporation by design of the intrinsic oculomotor behavior to implement gaze contingency, like the Alpha IMS and AMS devices. An additional crucial advantage in contrast to the Alpha IMS device is that there is no surgically maintained tether to an extraocular power supply because power is delivered as part of the infrared projection through the ocular optics, which significantly reduces the surgical risk.

Some limitations of the Alpha IMS and the Stanford approach have been recently addressed in a study that used polymer-based (rather than silicon-based), organic photodiodes to manufacture a subretinal prosthesis (Maya-Vetencourt et al., 2017). This photovoltaic approach has been tested in animal models with promising results. It offers high biocompatibility, as the post-mortem analysis showed that the prosthesis embedded in the tissue remained intact even after 6 months of implantation. Additionally, it offers light sensitivity close to the range of normal daylight illumination, as shown by the visually evoked potentials in response to dim flash stimuli of 20 cd m^-2^ (Maya-Vetencourt et al., 2017).

#### Limitations of the Retinal Approach

An important limitation of the retinal approaches - both intraocular and camera-connected devices - is that they require healthy retinal ganglion cells, and thus normally are considered viable treatments only for blindness due to degeneration of the photosensitive cells. Even in cases where degeneration is limited to the photosensitive layer, the retina exhibits remodeling of the remaining layers in response to loss of afferent input (Jones and Marc, 2005; Jones et al., 2016). Indeed, as a part of the disease process, ganglion cells — a critical target of retinal prostheses that activate this layer either directly (e.g., epiretinal devices; see Table 1) or indirectly (e.g., subretinal devices; see Table 1) — might also become compromised by, for example, higher spontaneous activity levels that are accompanied by higher cellular response thresholds to electrical stimulation compared to the healthy retina (Marc et al., 2003; Suzuki et al., 2004; Jones and Marc, 2005; O’Hearn et al., 2006; Sekirnjak et al., 2009; Chan et al., 2011). Additionally, there is some evidence that retinal implants may result in further or more aggressive remodeling (Butterwick et al., 2009). Remodeling processes complicate and perhaps contraindicate the retinal approach. The clinical implications of remodeling are also found in studies reporting that some epiretinal prosthesis users report unstable phosphene (e.g., phospene patterns perceived by the implanted patients do not always correspond to the geometric pattern of the electrical stimulation; Humayun et al., 2003), possibly due to ganglion cell loss and anomalous rewiring within the retina (Marc et al., 2003). These findings raise the question of whether future retinal implants with improved spatial resolution would be able to convey additional detail, or would be limited due to the pathological state of the retina in end-stage disease (Marc et al., 2003). Additionally, diseases such as glaucoma, diabetic retinopathy or ocular trauma compromise the function of the ganglion cell layer (Shepherd et al., 2013), thus limiting the applicability of retinal stimulation to restore vision.

### Non-retinal Approaches

The thalamic and cortical stimulation approaches seem to be better suited for diseases such as glaucoma or diabetic retinopathy, given that both the LGN and the primary visual cortical areas remain largely intact even with damage to the ganglion cells (Gupta et al., 2006; Gupta et al., 2008). Utilizing a quantitative positron emission tomography (PET), a recent study showed that both photic and electrical stimulation yield visual cortex activation in both retinally degenerated participants and healthy controls (but level of activation was lower in the diseased group; Xie et al., 2012). Although evidence suggests an association of neural degeneration in optic nerve, LGN, and visual cortex with clinical findings (Gupta et al., 2006), it remains unknown whether functional effects are manifested in the LGN or visual cortex as the disease progresses (Gupta et al., 2008).

#### Thalamic Approach

The LGN has been suggested as a candidate target for stimulation since it would provide treatment for a wide range of diseases including retinitis pigmentosa, age-related macular degeneration and ocular trauma (Pezaris and Reid, 2007, 2009; Pezaris and Eskandar, 2009; Bourkiza et al., 2013; Vurro et al., 2014; Killian et al., 2016). Thalamic microstimulation has been reported to generate phosphenes that were readily integrated into a visual task (Pezaris and Reid, 2007). In contrast with the retinal prostheses, the LGN approach carries several benefits, including the potential for high-resolution artificial vision due to foveal magnification; compared to cortical approaches, it reduces the surgical risks due to smaller craniotomies, while maintaining the advantages and the stability of the implant due to the intra-cranial location (Bourkiza et al., 2013). However, as with cortical prostheses (for a review on cortical prosthesis see Lewis et al., 2015), the thalamic approach uses an external camera that requires image compensation for gaze direction, since the RFs of LGN cells are retinotopically locked (Hubel and Wiesel, 1962; Wiesel and Hubel, 1966). Therefore, such devices must also include a mechanism that delivers stimulation that will compensate for changes in eye position, either by physically re-aiming the camera to point where the gaze is directed, or by electronic translation of the image (Pezaris and Eskandar, 2009). Proponents of the LGN approach are already working on gaze-contingent paradigms that will allow a better understanding on how gaze compensation can be integrated with a visual prosthesis (Bourkiza et al., 2013; Vurro et al., 2014; Killian et al., 2016).

#### Cortical Approach

The early visual cortical areas appear to be attractive candidates for a visual prosthesis, since they allow for the possibility to implant a large number of electrodes that could potentially offer high-resolution vision (Lewis et al., 2015). Specifically, the organization of area V1, its large surface area, and foveal magnification have led researchers to propose cortically based devices as promising stimulation targets (Troyk et al., 2003; Schiller and Tehovnik, 2008). Additionally, cortical function remains intact in most blindness-related diseases, thereby enabling restoration of vision despite degenerations of retinal ganglion cells or optic nerve injury. The pioneering work of Brindley and Lewin (1968) was the first important demonstration of implanting electrodes over the visual cortex to evoke artificial visual percepts. However, given the complex physical structure and functional organization of the cortex, most stimulation attempts in both humans and animal models thus far have yielded inconclusive results (Dobelle, 2000; DeYoe et al., 2005; Murphey et al., 2009; Tehovnik and Slocum, 2009; Torab et al., 2011; Beauchamp et al., 2012; Lewis et al., 2015). It remains to be seen whether the Orion cortical prosthesis (Second Sight), implanted in the first patient after receiving conditional FDA approval in August 2017, will provide more robust and accurate artificial percepts.

## Dealing With Eye-Camera Misalignment

To a great extent, the efforts of restoring vision have been focusing on issues other than gaze contingency. Yet, to provide a viable solution to blind individuals, the issue of gaze contingency needs to be investigated in more detail. Without compensation for gaze direction, the visual prosthesis device is less likely to be a positive assistance for the patient’s daily activities. Thus, it is important to explore ways to efficiently and accurately update the artificial percepts based on gaze direction.

To address the effect of eye-camera misalignment on spatial localization, Sabbah et al. (2014) had patients implanted with the Argus II device shift their gaze toward different locations on a screen, while maintaining their head still. After each gaze shift, they had to report the perceived target location by pressing with their finger on a touch screen at the corresponding spot. When patients voluntarily shifted their eyes (i.e., with the camera mounted on the glasses) while pointing at a light source, the locations to which they pointed were deviated toward the direction of the gaze. Thus, the misalignment between the head (i.e., camera position) and the participants’ gaze interfered with their perception of spatial localization, thereby affecting visuomotor coordination. The interference was consistent with what we would expect based on extensive evidence that phosphenes are encoded in retinotopic space (e.g., Brindley and Lewin, 1968; Dobelle and Mladejovsky, 1974; Andersen et al., 1985; Gauthier et al., 1990; Schmidt et al., 1996; Veraart et al., 1998; Pezaris and Reid, 2007; Caspi et al., 2017), and our theoretical understanding of the early visual system (e.g., Hubel and Wiesel, 2004).

Eye-camera misalignments may be exacerbated due to the common oculomotor abnormalities observed in blind individuals (Schiller and Tehovnik, 2008). Recent attempts aimed to investigate whether the mechanisms underlying oculomotor functioning remain intact in blind patients by examining whether they can adapt to shifts or distortions in their percepts as has been shown in sighted individuals when wearing prism glasses (Gibson, 1933; Held and Hein, 1958; Redding and Wallace, 1988; Buch et al., 2003; for reviews see Hopp and Fuchs, 2004; Herman et al., 2013). Such adaptations in normals are crucial in correcting both localization and coordination errors. To assess whether patients with camera-connected prostheses could also adapt to misaligned percepts, Barry and Dagnelie (2014, 2016) introduced camera misalignments that ranged 15 to 40° from optimal camera alignment position. Interestingly, two of three patients exhibited significantly increased accuracy during the period in which they were presented with camera misalignments with a remarkably slow average rate of 0.02°/day (approximately 4000 times slower than the rates seen in sighted participants adapting to prism glasses, and apparently not consistent with other adaptive experiments), while the improvement was highly dependent on the presence of auditory feedback. Despite the limited improvements reported by Barry and Dagnelie (2014, 2016), further studies have provided more optimistic results by showing that, even in the presence of blindness-related oculomotor abnormalities, saccadic accuracy can be plastically re-trained even in individuals suffering from RP (Ivanov et al., 2016) and AMD (Kuyk et al., 2010), thus indicating that eye-tracking might be effective in individuals with oculomotor abnormalities, once the appropriate oculomotor training regimes are introduced. These findings highlight the effects of camera misalignment to perceived location of the stimuli in patients implanted with a camera-connected prosthesis without gaze compensation. Integrating gaze-contingent information to the percept provided by the camera would, therefore, contribute in overcoming this dissociation between camera (i.e., head) and eyes and consequently in eliminating localization errors made by patients in their attempt to reach or grasp an object.

### Oscillopsia as a Clinical Analogy

Oscillopsia (Brickner, 1936) is a set of disease conditions that create the impression of visual instability and, through understanding of these illusions, can inform the design of visual prostheses. The primary diseases in oscillopsia are nystagmus and vestibular areflexia, characterized broadly by unintended ocular motion that leads to decreased visual acuity, nausea, and vertigo (Tilikete and Vighetto, 2011) that can be debilitating (Evans, 1989). Causes of oscillopsia are typically brain-stem and cerebellar lesions due to stroke, tumor, or multiple sclerosis (Bronstein, 2013). The externally observable symptoms are frequent deflection of the eyes from fixational gaze, often appearing to be periodic or oscillatory. While the mechanisms of oscillopsia remain incompletely explored in animal models (e.g., Dicke et al., 2008; Dash et al., 2009; Subramaniyan et al., 2013) and clinical settings (reviewed in Straube et al., 2004), the primary hypothesis is that the discordance between intended eye position and the actual position of the ocular plant is the root cause of the cognitive and visual effects. It is additionally hypothesized that it is the discordance itself rather than its oscillatory nature that underlies the visual malady (Gresty et al., 1977), suggesting that any persistent lack of correspondence between the eye position and its cortical representation may result in similar effects. Since such dissonance exists in visual prostheses based on external cameras without gaze-compensation — there is generally a lack of correspondence between the position of the imaging apparatus, being the camera, and the position of the ocular plant as still represented in the cortical pathways — we might accordingly expect malperception to result.

## Eye-Tracking: a Viable Solution?

The dissociation between retinally steered phosphenes and the comparably static image captured by an external camera may be addressed by integrating an eye-tracker to the visual prosthesis (Bourkiza et al., 2013; Vurro et al., 2014; Caspi et al., 2017; Rassia and Pezaris, 2018). Compensation may be obtained by electronically shifting the image obtained from the camera on a frame-by-frame basis by the instantaneous gaze position prior to deriving the stimulation patterns for the phosphene locations in the visual field (Pezaris and Eskandar, 2009). By using an eye-tracker, Caspi et al. (2017) assessed whether the brain of patients implanted with the retinal prosthesis can map stimuli that had not been compensated for gaze position from retinotopic to world-centered coordinates. To measure the accuracy of the mapping, the retina was directly stimulated via the Argus II device and the location of the resulting percept in world-coordinates was recorded. However, given that the calibration stage required in eye-tracking experiments depends on the subject’s gaze being directed to predefined points in space (typically shown on a computer monitor), this procedure currently cannot be done accurately with blind individuals. Inspired by previous studies that have proposed the usefulness of pointing methods for mapping phosphenes (Brindley and Lewin, 1968; Everitt and Rushton, 1978; Dobelle et al., 1979; Veraart et al., 1998), Caspi and colleagues (2017) used a mobile eye tracker and a pointing method to examine whether blind individuals can map the percept of a retina-centered electrical stimulation to the correct location in world-centered coordinates. At the beginning of each trial, the patients were asked either to look straight ahead or move their eyes to the right or left. This procedure allowed the experimenter to observe the real-time pupil image and to ensure that the pupils were aimed at the requested position. Subsequently, one out of three groups of electrodes was selected for stimulation that lasted 600 ms. After the offset of the stimulation the patients were asked to place a handheld marker at the location of the elicited phosphene, providing the location of their percept in world-centered coordinates. Interestingly, with retinocentric (i.e., gaze-contingent) electrical stimulation patients were able to locate the percept correctly in head-centered coordinates, thus again verifying that the brain accurately shifts the artificial percept based on the position of the eye. This, in turn, demonstrates that an eye-tracker can be coarsely calibrated on blind patients based on the percept from the implant (Caspi et al., 2017).

Despite the promising results from Caspi et al. (2017), some important issues need to be reviewed. First, this study did not include a gaze-contingent mechanism, that is, the stimuli were presented without compensation for eye position, a decision made to ensure the relevance of their results to ongoing work. Second, the patients that participated in this study had retinitis pigmentosa but as reported in the paper, they did not exhibit any abnormal oculomotor behavior such as nystagmus or strabismus. Evidence suggests that the late stages of the disease can be accompanied by severe oculomotor abnormalities, with most adult-onset blind patients with RP exhibiting uncoordinated, multidirectional, and disjunctive eye movements or persistent nystagmus (Cohen, 2007; Schiller and Tehovnik, 2008). These abnormalities might limit the ability to calibrate an eye tracker and have been, thus, implicated as a major problem in clinical deployment of a prosthetic device (Schiller and Tehovnik, 2008). Additionally, even in the absence of oculomotor abnormalities, eye tracking may not be applicable in patients with impaired functioning of the eye muscles (Lewis et al., 2015). Given that neither the age of the patients nor the disease onset is addressed by Caspi et al. (2017), it remains unknown whether the findings are applicable to older blind individuals with late-stage disease.

Eye-tracking has been recently used in an attempt to characterize the oculomotor behavior of implanted patients with the subretinal intraocular device, Alpha IMS (Hafed et al., 2016). The two patients in this study were presented with geometric shapes with luminances of either 97 cd/m^2^ (bright) or 28 cd/m^2^ (dim) on a dark background and they had to report seeing them or not, by pressing a button for as long as they had a percept of a stimulus and release it when that percept disappeared. By tracking the patients’ eye movements and comparing them to those of three healthy controls, the study showed that once the patients localized the shapes, their fixational patterns were reminiscent of those of the controls, i.e., they generated microsaccades and ocular drifts. Additionally, the study reported a correlation between loss of stimulus visibility, as estimated by button press duration, with reductions in frequency of saccades and microsaccades. More importantly, gaze location corresponded to the location where the stimulus was presented, while also shape and size characteristics of the presented stimulus were reflected by the direction and size of saccades providing evidence of accurate visual exploration. These findings highlight the importance of using eye tracking both as a diagnostic and a training tool in implanted patients, since it would allow (a) measurement of oculomotor behavior when provided with artificial percepts, an objective metric for evaluating implant performance and (b) development of well-designed oculomotor training paradigms.

The use of eye-tracking has been suggested for prosthetic vision (Sommerhalder et al., 2004; Pezaris and Reid, 2007; Perez Fornos et al., 2011; Hafed et al., 2016) and examined more recently for the thalamic approach in particular (Marg and Dierssen, 1966; Chapanis et al., 1973; Bourkiza et al., 2013; Vurro et al., 2014; Killian et al., 2016). Inspired by an understanding of the fundamental retinotopic-to-spatial coordinate mapping performed by the visual system (Gnadt et al., 1991; Andersen et al., 1993), some artificial vision studies have implemented gaze-contingent display paradigms such as moving window (Richlan et al., 2013) or related mechanisms (Bourkiza et al., 2013; Vurro et al., 2014; Rassia and Pezaris, 2018). During these tasks, the display changes in response to the participant’s eye movements in a real-time fashion with minimum possible latency (Richlan et al., 2013). Importantly, to create artificial percepts close to those of natural vision, the image obtained by the device’s imaging sensor needs to adapt instantaneously during saccadic eye motions in order to simulate saccadic suppression, the transient silencing of neural activity during rapid eye movements.

Saccadic suppression has been extensively studied, but remains enigmatic (for reviews see Ross et al., 2001; Burr, 2004; Ibbotson and Krekelberg, 2011). Saccadic suppression does not appear across all visual brain areas (Thilo et al., 2003) and is differentially sensitive to varying stimulus characteristics (Burr et al., 1994; Bridgeman and Macknik, 1995). For example, suppression is selective to low frequency luminance modulation with rapid on/off transitions, suggesting that only magnocellular function is suppressed, while parvocellular function remains unaffected (Burr et al., 1994). Additionally, phosphenes elicited by retinal stimulation are suppressed during a saccade, whereas phosphenes induced by transcranial magnetic stimulation to the occipital cortex are unaffected (Thilo et al., 2003). This latter finding provided the first strong evidence that the mechanism underlying saccadic suppression operates at an early stage of the visual pathway, possibly within the LGN or the primary visual cortex (Thilo et al., 2003). Overall, these findings have important implications for visual prostheses depending on the stage of the visual pathway they are targeting and argue in favor of prosthetic devices that could incorporate or simulate saccadic suppression.

Although eye-tracking allows the implementation of gaze-contingency in both simulation paradigms and clinical studies with implanted patients, its practical limitations need to be considered (Bourkiza et al., 2013; Richlan et al., 2013). Crucially, the eye-tracking methodology is often accompanied by inherent noise and system latency. This latency results in increased spatial noise in the images, which may affect participants’ performance in simulation studies (Keesey, 1960; Westheimer and McKee, 1975). Beyond its implementation in simulation tasks, eye-tracking has been also used in studies aiming to analyze the eye movements in implanted patients for investigational purposes (Hafed et al., 2016). However, eye trackers typically require infrared illumination to detect the pupil and corneal reflection in order to track gaze location. This makes it particularly challenging to examine the oculomotor behavior of patients implanted with devices using photodiodes (e.g., Alpha IMS or AMS), since they are also sensitive to IR radiation, which could interfere with participants’ percepts (Hafed et al., 2016). To avoid this problem, it has been proposed to use an occluder between the implanted and non-implanted eyes and instruct the patient to look with the implanted eye at the stimulus, while the eye tracker would illuminate the non-chip eye (Hafed et al., 2016). Thus, in this case, all eye-tracking measurements would be obtained from the non-implanted eye (Hafed et al., 2016).

In addition to all these issues, studies using eye-tracking to test device efficacy need to take into account the oculomotor patterns of implanted patients and the similarities and/or differences they exhibit compared to the eye movements of sighted individuals. For example, while in sighted individuals the eyes move from one location of the visual scene to another approximately 2 to 3 times per second (Fischer, 1992; Ross and Ma-Wyatt, 2003; Burr, 2004; Ibbotson and Krekelberg, 2011), many blind patients exhibit several oculomotor abnormalities, such as uncoordinated eye movements or persistent nystagmus (Cohen, 2007; Schiller and Tehovnik, 2008). Additionally, while reading, sighted individuals exhibit mean fixation time around 200–250 ms and saccadic durations of 20–35 ms (Richlan et al., 2013). Interestingly, patients implanted with Alpha IMS have been found to exhibit fixational eye movements that were similar to those made by sighted control participants (Hafed et al., 2016). Saccades made by the patients when they reported perceiving a stimulus were significantly smaller as compared to when they could not see the stimuli, which has been suggested to indicate the level of fixational stability (Hafed et al., 2016).

Taken together, eye-tracking holds the premise of being a crucial step forward in artificial vision studies. Integrating a wearable eye tracker with an external-camera visual prosthesis will contribute to providing the implanted patients with accurate artificial percepts, thereby enhancing the ability to carry out tasks of daily living. At the same time, eye tracking should provide insight into oculomotor characteristics in blind people, including device-specific temporal properties of saccadic reactions (Hafed et al., 2016; Caspi et al., 2017). Using a basic behavioral response like eye movements provides a quantifiable, objective means to measure device utility that can be paired with subjective patient observations to, in turn, assess and optimize prosthesis efficacy.

## Future Directions

Gaze contingent updating of visual stimulation seems to be an important challenge for visual prosthetics research. The main objectives of this paper were to review (a) the evidence about the mechanisms underlying spatial updating and (b) the implementation of these findings to artificial vision studies. The fact that gaze compensation occurs in cortical areas beyond the primary visual cortex in the visual processing stream creates the need to take into account this cortical feedback when designing future visual prosthetic devices. Although the mechanism underlying spatial updating is not yet fully understood, artificial vision studies have already acknowledged the importance of gaze compensation.

Eye-tracking methodology has been suggested as a possible solution to overcome the hurdle of gaze contingency. While some of the devices introduce intraocular light-sensing (Zrenner, 2002; Stingl et al., 2013a,b, 2015, 2017; Lorach et al., 2015), other approaches are currently working on updating the artificial percept based on the patient’s eye movements by integrating an eye tracker with the visual prosthesis (Bourkiza et al., 2013; Vurro et al., 2014; Caspi et al., 2017). This will improve the patients’ quality of life by increasing their independence during ADL. Gaze contingent paradigms will shed light into the brain circuitries of visual mapping while they will also allow comparing the mapping mechanisms in sighted and blind individuals using a visual prosthesis.

**Table 2:** Questions for future research.

What quantitative advantages do fully gaze-contingent systems have over non-gaze contingent ones?
How should we account for saccadic suppression when providing electrical stimulation?
What are the oculomotor differences between sighted and blind individuals?
Are abnormalities extinguishable with reintroduction of functional sight?
Is rigorous rehabilitation required? Given that some of the diseases causing blindness affect the aging population (RP, AMD, glaucoma), should we consider age-related changes in oculomotor characteristics such as reduced saccadic accuracy and decreased vestibulo-ocular function?
Will the decreased vestibulo-ocular function recover under treatment with a gaze-contingent visual prosthesis that does not specifically target the accessory optic system?
Attempts to artificially restore the VOR (i.e., amplitude modulated electrical stimulation of the ampullary branches of the vestibular nerve) have reported that gaze stabilization mechanisms in patients with bilateral vestibular loss can be restored using a vestibular implant (Will the use of eye-tracking technology be applicable in visual prosthetics given the uncoordinated and compromised eye movements observed in blind individuals?
Will oculomotor training paradigms successfully re-establish saccadic accuracy in blind patients with uncoordinated eye movements?


Further research needs to be conducted on the effects of head vs. eye movements on processing visual information in different types of ADL, including reading, navigation and object recognition. Investigating the contribution of both head- and eye-movements would provide a better understanding of the gaze patterns and visual scanning strategies adopted by patients implanted with camera-connected devices. Addressing this question is of critical importance, since to-date, prosthetic devices do not provide spatially continuous visual information, which leads to incoherent percepts of isolated discrete phosphenes. Visual scanning is necessary to fill in the lack of information. For example, although a static image of phosphenes may appear as discrete and disjointed dots, they are integrated into more coherent structural percepts, once the person initiates movements to scan the visual scene (Chen et al., 2006, 2007). This critical enhancement of visual acuity is why head movements have been reported to be essential for implanted patients, since they support visual exploration (Chen et al., 2006, 2007; Dobelle, 2000).

The brain possesses an inherent ability to adopt alternative scanning strategies to process the visual scene (Gilchrist et al., 1997; Chen et al., 2006, 2007). For example, in the study by Gilchrist et al., 1997, a patient without the ability to perform ocular motions performed head scanning of the scene in a way reminiscent of the patterns of natural eye movements, suggesting that the saccade-like head movements are the optimal sampling method adopted by the brain under those conditions. The importance of head movements has been also demonstrated by artificial vision simulation studies that explored the head scanning behavior when the subjects had to complete a task with their eye movements being restricted (Chen et al., 2006, 2007). To investigate the profile of several head movement metrics (i.e., displacement, velocity, and acceleration), Chen et al. (2007) introduced a simulated prosthetic vision paradigm using a head-mounted display, where participants viewed the visual stimuli of a standardized Landolt C test with their right eye only. Using a head tracker, each frame was updated based on participants’ head movements, which allowed them to scan the stimuli. Importantly, feedback was provided after each response to facilitate learning. As expected, increased head movements were observed when performing the visual acuity task in simulated prosthetic vision sessions as compared to the control trials (Chen et al., 2007), thus highlighting their role in centering and scanning the test stimuli. Interestingly, in agreement with Gilchrist et al. (1997), the head displacement profile, and in particular the delay onset to head movement was reminiscent of that of saccades performed by the eye. Thus, even in the absence of pathological conditions (e.g., Gilchrist et al., 1997), with restricted eye movements, the brain calls upon the head to compensate. More importantly, the increases in head velocity were significantly correlated with visual acuity performance, indicating that increased scanning velocity increases the sampling rate of the phosphene patterns (Chen et al., 2006, 2007). This was further supported by a modulation of head movement by task demands: scanning velocities increased with increasing cognitive load. However, an important limitation of these studies was the absence of eye-tracking, which would provide a better understanding of the combined head-eye scanning patterns and how they are related to performance outcomes (Chen et al., 2006, 2007).

Taken together, the flexibility of the brain to adopt alternative strategies to optimize visual acuity creates the need to explore re-training of gaze shift accuracy through either movements of the head or of the eyes in everyday tasks. Simulation studies have already started to explore the scanning methods adopted by the brain to process the visual scene (Chen et al., 2006). Further studies are, though, needed to explore which scanning strategy would be more efficient for the implanted patients to extract visual information from the scene. Understanding these strategies would contribute significantly in post-implantation rehabilitation to guide patient adoption of scanning patterns appropriate to task characteristics. It is currently unknown whether the findings reported by gaze-contingent simulation studies (e.g., Bourkiza et al., 2013; Vurro et al., 2014; Rassia and Pezaris, 2018) would be replicated in a similar head-contingent simulation task. The plasticity reported by Chen et al. (2006, 2007) suggests that with the appropriate amount of training and with the presence of useful feedback, participants might be able to learn to perform everyday tasks by using their head-movements only. However, given that head movements are slower than eye movements (e.g., Freedman and Sparks, 1997), an important difference between the two paradigms would be the time required to complete the task (e.g., reading speed), consistent with previous findings (Gilchrist et al., 1997). All these questions are highly relevant both for current and for future prosthetic devices, as well as for the post-implantation rehabilitation plans that could assist the patients in making use of the new artificial visual signals provided by the implant.

## Conclusion

Overall, the studies on visual prosthesis paint an optimistic picture of the future of artificial vision, and thus of the improvements in quality of life that could be offered to blind patients. However, the field needs to address the challenging issue of gaze contingency in order to allow prosthetic devices to provide robust artificial visual perception. We, thus, believe that several questions remain unanswered (see Table 2) and thus warrant further investigation in order to create cutting-edge prosthetic devices to substantially contribute to the everyday life of the blind population.

## Author Contributions

NP performed the literature review, analyzed the current state of the field, and wrote the majority of the text. JP provided the advice and guidance and assisted in writing the text.

## Conflict of Interest Statement

The authors declare that the research was conducted in the absence of any commercial or financial relationships that could be construed as a potential conflict of interest.

## Glossary

### Activites of Daily Living (ADL)

Normal activities such as grooming, dressing, eating, moving through familiar and/or unfamiliar environments, handling money, recognizing objects and faces, reading, etc.

### Age-Related Macular Degeneration (AMD)

A disease that causes damage to the macula, the area near the center of the retina that allows us to see fine detail. AMD blurs the sharp, central vision needed for activities like reading and driving.

### Camera-Connected Devices

Prostheses that generate artificial visual percepts based on external cameras typically built into glasses worn by the patient. A limitation of these devices is that camera direction is typically determined by head position alone and not updated for the eye movements.

### Choroid

A tissue layer located between the retina and the sclera, containing a rich network of blood vessels and connective tissue that is responsible for providing oxygen and nutrients to the outer layers of the retina.

### Cones

A class of photoreceptor cells in the outer retina, responsible for color vision, and optimized for bright illumination (like daylight environments). Cones are densely packed in the central part of the retina, but are relatively sparse in the periphery. The human eye has approximately 6 million cone cells.

### Corollary Discharge (or Motor Efference Copy)

The hypothesized copy of a movement command signal originating in a sensorimotor area that is made available to other, non-motor areas. This copy is sent to regions of the brain that require information about the impending movement rather than being involved in generating or controlling the movement, and is thought to help interpret ongoing sensory input from the eyes, or plan future actions.

### Epiretinal Implants

Implants placed in the vitreous cavity directly over the inner retinal surface in close proximity to the ganglion cell bodies.

### Extra-Retinal Information

Information about the current visual perception that does not originate in the retina. Examples include sensory feedback from the muscles and efferent motor information.

### Fixation

The close visual examination of a target with the eyes held at a single position. Fixations typically last a few hundred milliseconds, after which a saccade (q.v.) takes the gaze location to a new fixation. Fixations typically happen two or three times per second. Contrast with smooth pursuit (q.v.).

### Fixational Eye Movements

Involuntary eye movements that occur during fixation. During a fixational period, the eyes are not entirely still, but exhibit small movements called microsaccades, slight drifts and tremor. Fixational eye movements are not typically observable without instrumentation.

### Fovea

A region of the macula (q.v.) responsible for sharp central vision, typically capturing the central 1–2 degrees of vision. Characterized by the highest density of photoreceptors in the retina. Distinguished by the exclusion of cells other than cone photoreceptors, a substantially different architecture than the remainder of the retina. When we speak of looking at, or visually capturing, a target, the fovea will be imaging the target to examine it with the highest available acuity.

### Gaze-Contingency

The dynamic updating of a visual presentation based on the position of the observer’s gaze. A major challenge in many current prosthetic devices is to update the content of artificial percepts based on the instantaneous gaze location, allowing the user to naturally view using normal eye movements. Contrast with head-steering (q.v.).

### Head-Steering

The aiming of a scene camera by movements of the head, and not the eyes. Such head movements are often called scanning motions and result in much slower examination of a visual scene than when eye motions are incorporated into the steering mechanism.

### Intraocular Light-Sensing

The prosthesis designs with an array of photosensing circuitry implanted on the retina that use existing ocular optics to capture the visual scene. These approaches automatically update the intraocular imaging sensor with eye movements and are thus relieved of the burden of separately implementing gaze contingency.

### Landolt-C

A standard clinical test for measuring visual acuity. The Landolt C is a broken circle in which the width of the gap and the stroke width are both equal to one-fifth the height of the circle. The gap appears at one of four locations, which the subject indicates by reporting “right,” “down,” “up,” or “left.” With the gap to the right, the figure resembles a capital letter C. The size of the gap of the smallest figures whose positions can be reliably reported is equivalent to the MAR (q.v.).

### Lateral Geniculate Nucleus (LGN)

A deep, central structure, which is the main relay center in the brain for visual information coming from the retina. The axons of the LGN project directly to the visual cortex. The LGN is the only location within the early visual system where the three major pathways (magnocellular, parvocellular, and koniocellular) are macroscopically segregated. Thus, it may be the only location supporting selective stimulation of the three pathways, and thus independent control of luminance and chrominance information.

### LogMAR

See MAR.

### Macula

The small, sensitive area of the retina that provides central vision. It is located in the center of the retina and contains the fovea.

### Minimum Angle of Resolution (MAR, logMAR)

The minimum feature size reliably seen by an individual with or without optical correction of natural sight. Expressed as an angle in the visual field, and is therefore independent of distance. Equivalent to the gap/stroke width in a Snellen E, or the gap/stroke width in a Landolt C. The logMAR is the base-10 logarithm of MAR, and is typically used to report visual acuity. Normal vision (Snellen 20/20 or 6/6) is equivalent to logMAR 0.00. Values above 0.00 are worse-than-normal acuity, and values below 0.00 are better-than-normal.

### Microsaccades

Small, involuntary saccades produced during fixation. Microsaccades shift the retinal image in a way that prevents adaptation or visual fading.

### Motor Error

The angular movement in the visual field through which the eyes must move to correctly foveate an object. This vector begins equal to the retinal error caused by the location of the stimulus prior to a gaze shift (q.v.), and requires updating for any intervening eye motions.

### Nystagmus

A condition of repetitive, rapid, and uncontrolled eye movements, which is often observed in blind patients. This oculomotor abnormality (q.v.) is further categorized into horizontal, vertical, and rotary nystagmus depending on the direction of the eye movements (i.e., side to side, up and down, or in a circle, respectively).

### Oculomotor Abnormalities

Pathologies linked to blindness often manifested as persistent nystagmus or uncoordinated, multidirectional, and disjunctive eye movements. They are more pronounced in congenitally blind individuals than in those who become blind later in life.

### Optic Nerve

A bundle of about one million nerve fibers that transmits visual information from the retina to the brain and is part of the central nervous system. Formed by the axons of the retinal ganglion cells (q.v.).

### Oscillopsia

An oculomotor abnormality (q.v.) often resulting from impaired vestibulo-ocular reflex (q.v.), where objects appear to bounce because they do not remain fixed at the same location on the retina.

### Phosphenes

Visual percepts that are not produced by light. Phosphenes can be evoked by electrical, mechanical, or magnetic stimulation of the retina, optic nerve, LGN or visual cortex.

### Photoreceptors

Neurons in the retina that transduce photons to neural signals, with two primary types, cones (q.v.) and rods (q.v.). Many diseases of the eyes that cause blindness interfere with the normal activity of photoreceptors.

### Receptive Fields (RF)

The region of sensory space whose stimulation directly results in the response of a neuron. Thus, the part of the visual field, or the region of space from which photons can excite a single neuron, describes that cell’s receptive field. Synonym with **Response Field**.

### Retinal Error

The distance between the retinal stimulation caused by a target and the fovea (q.v.). To foveate an object of interest, the retinal error must be brought to zero.

### Retinal Ganglion Cells (RGCs)

Nerve cells whose axons connect the retina to the brain via the optic nerve. There are approximately 1 million RGCs in a normal human retina. RGCs can be stacked five layers deep surrounding the fovea, whereas in the peripheral retina, they form just a single layer. This eccentricity-dependent architectural stacking makes selective activation of foveal vision RGCs challenging for retinal implants.

### Retinal Prostheses

Devices that stimulate the retina in order to restore vision to the blind. Retinal prostheses are classified according to the part of the eye that is stimulated, epi-retinal (q.v.), sub-retinal (q.v.), or choroidal (q.v.).

### Retinal Remodeling

A consequence of retinal degenerative disease, characterized by morphological, biochemical and physiological alterations, resulting in hyperactive RGCs (q.v.), which exhibit spontaneous, oscillatory bursts of spikes. This aberrant electrical activity degrades the signal-to-noise ratio in the cells’ responses, and consequently the quality of information they send to the brain. Retinal remodeling poses major challenges for retinal approaches.

### Retinitis Pigmentosa (RP)

A genetically based, degenerative eye disease caused by an abnormality of photoreceptors that leads to peripheral blindness. People suffering from RP experience night blindness and a progressive loss of the peripheral visual field, eventually developing tunnel vision.

### Retinotopic Mapping

The orderly visual map of the retina, reproduced in structures of the central visual system including LGN and visual cortex. These structures carry a representation of the visual image such that adjacent regions of the image are represented by neighboring regions of the visual area. Just as the neural activity across the retina represents an image projected on retinal photoreceptors, the neural activity across other visual areas equivalently represents the image in a spatially continuous form.

### Rods

A class of photoreceptor cells (q.v.) that is responsible for vision at low light levels such as found under starlight. Rods are not color-sensitive and have low spatial acuity. Normal day-time vision is primarily from cones (q.v.), rather than rods.

### Saccades

Swift point-to-point eye movements that occur between periods of fixation (q.v.). Each saccade causes rapid motion of the retinal image and delivers a different view of the world to central vision.

### Saccadic Suppression

The transient reduction in sensitivity to visual stimuli during saccades. The mechanisms and reasons for saccadic supression are not completely understood. The LGN may have a critical role in mediating the effect.

### Serious Adverse Event (SAE)

Medical occurrences that are life-threatening or result in death. Such events result in permanent impairment of a body structure or function, prolonged hospitalization, or required medical or surgical intervention.

### Smooth Pursuit

Tracking eye movements used to stabilize the image of a moving target on the fovea.

### Snellen Acuity Test

A standard test of visual function using a chart of letters in decreasing font size. The smallest line a subject can read yields their visual acuity and is expressed as a fraction, e.g., 20/20 (in Europe, 6/6). The numerator refers to the distance from the subject to the chart (20 feet or 6 m) and the denominator is the distance at which a person with no impairment would be able to see the same line. Normal vision is 20/20 (or 6/6), worse-than-normal acuity has larger numbers in the denominator, better-than-normal has smaller numbers. See also MAR, logMAR.

### Spatial Updating

The process of combining retinal signals with extra-retinal information that supports spatial constancy to provide a continuous, stable representation of visual space. Without spatial updating, the visual input to the brain appears to shear radically with each eye movement, severely limiting interpretability.

### Strabismus

An oculomotor abnormality (q.v.) often observed in blind patients, in which the eyes are not aligned properly and point in different directions. Typically, one eye looks straight ahead, while the other turns inward, outward, upward, or downward.

### Subretinal Implant

Implants placed in the region between the photoreceptor layer and the pigment epithelium known as the subretinal space.

### Superius Colliculus (SC)

A midbrain, retinotopically organized structure that receives inputs from the retina and visual cortex at its superficial layers, and multisensory inputs at its intermediate layers. Involved in generating and controlling voluntary saccades.

### Suprachoroidal Implants

Implants placed in the region between the sclera and the choroid, located toward the external surface of the eye. Typically exhibit lower resolution that subretinal or epiretinal implants due to the increased distance from the RGC layer.

### Vestibulo-Ocular Reflex (VOR)

An automatic mechanism that contributes in stabilizing gaze during head rotations by producing an eye movement in the direction opposite to the head movement. This function often exhibits impairments in blind individuals.

### Visual Cortex

Areas of the cortex devoted to processing visual information. The primary visual cortex is the first stage of conscious vision, is located in the occipital pole, and receives input from the LGN.

### Visual Prostheses

Devices designed to provide artificial vision for the blind, which are classified according to the brain area they target for stimulation, such as the retina, the LGN, or the cortex. Two major design variants use either an imaging apparatus placed within the eye and include the optics of the eye in their operation, or a camera on a set of glasses worn by the subject. Whether the imaging sensor is steered by the eyes typically determines whether or not the prosthesis provides gaze-contingent (q.v.) stimulation.
